# A practice already in use: a snapshot survey on the use of doxycycline as a preventive strategy (Doxy-PEP and Doxy-PrEP) in the GBMSM population in Spain

**DOI:** 10.1007/s15010-024-02320-y

**Published:** 2024-07-25

**Authors:** Sergio Villanueva Baselga, Mora Ruben, Villegas Luis

**Affiliations:** 1 Stop Sida, Barcelona, Spain; 2 Centre of Research for Information, Communication and Culture (CRICC), University of Barcelona, Barcelona, Spain

**Keywords:** Doxycycline, Sexually transmitted infections, Prophylaxis, PEP, Sexual health, Gay, bisexual and other men who have sex with men, GBMSM

## Abstract

**Purpose:**

. While doxycycline shows promise as a preventative measure against certain STIs (post-exposure prophylaxis or PEP, and pre-exposure prophylaxis or PrEP), very few medical and scientific associations favor its community use. Nevertheless, sexual health organizations for gay, bisexual and other men who have sex with men (GBMSM) community have noticed an increase in requests of its use.

**Methods:**

. The sexual health organization “Stop Sida” in Spain launched an anonymous snapshot survey to evaluate the current use of doxycycline as prevention strategy (both PEP and PrEP) in Spain through its social networks and its support groups in telegram.

**Results:**

. 150 valid answers were obtained from different parts of Spain. 82 respondents (54,6% of total) had ever taken doxycycline, out of which 29 (35,4%) indicated the use as doxy-PEP and 7 (8,5%) as doxy-PrEP. The self-reported rate of STI diagnoses (mainly of syphilis and chlamydia) in the past 12 months was lower among those participants who reported having used doxy-PEP compared with those who did not report using doxy-PEP. The most common ways to obtain the doxycycline were telling a specialist that they have had a risky contact or by using leftover pills from a previous treatment.

**Conclusion:**

The current study is the first study to describe the use of doxycycline as a preventive strategy among the GBMSM community in Spain, and the first designed, launched and analyzed entirely by an NGO dealing with sexual health for the GBMSM community. The results obtained are aligned with other studies in other parts of Europe, such as Germany.

**Supplementary Information:**

The online version contains supplementary material available at 10.1007/s15010-024-02320-y.

## Introduction

The growing incidence of bacterial sexually transmitted infections (STIs) is a major public health concern worldwide. Consequently, there is a need for new preventive strategies tailored to the needs of various social and vulnerable groups. In this regard, the efficiency of using doxycycline as a method of post-exposure prophylaxis (Doxy-PEP) against syphilis (*Treponema pallidum*), chlamydia (*Chlamydia trachomatis*) and, to a lesser extent, gonorrhoea (*Neisseria gonorrhoeae*), has been increasingly supported by evidence from several clinical studies conducted in recent years (such as IPERGAY in France in 2015 [1], Doxy-PEP in the US [2], and DOXYVAC [3] in France in 2022). These clinical trials have evaluated Doxy-PEP as a 200 mg dose taken within a maximum of 72 h after condomless sex. Conversely, the use of doxycycline as pre-exposure prophylaxis (Doxy-PrEP) has been less extensively studied, though it has shown promising results in smaller sample sizes [4].

Despite this evidence, very few medical and scientific associations favor its community use primarily due to concerns about potential antibiotic resistance. The European AIDS Clinical Society, in its 2023 guidelines update, indicates that doxy-PEP can be proposed to persons with repeated STIs living with HIV or taking PrEP for HIV only on case-by-case basis [5, 6]. Following these recommendations, several national associations in European countries have issued similar guidelines. For instance, the German STI Society advocates for a case-by-case recommendation [7]. Similarly, in Spain, the Spanish Association for Infectious Diseases and Clinical Microbiology (SEMIC) has taken a cautious stance, recommending Doxy-PEP only on a case-by-case basis and without indication [8]. However, there have been more prone positionings outside Europe. That is the case of the Australasian Society for HIV, Viral Hepatitis, and Sexual Health Medicine (ASHM) that, based on these results, stated in an interim position statement that doxy-PEP could be useful for people with repeated bacterial STIs [8].

These concerns from part of the medical and scientific communities have not, however, prevented the community of gay and bisexual men and other men who have sex with men (GBMSM) from including doxy-PEP and Doxy-PrEP as part of their prevention strategies. Some activist groups and sexual health NGOs, such as *TheLoveTank* in the UK, are actively increasing awareness among GBMSM community to include doxy-PEP as a preventive strategy for bacterial STIs. Nevertheless, there are few studies that have surveyed GBMSM community on how are they implementing such strategy. Examples of such studies have been performed in London in 2018 as a sub-study of IPERGAY trial [9], in Melbourne in 2019 [10], in the Netherlands in 2020 [11], or in Southern California in 2021 [12]. One of the most recent studies has been conducted in Germany by Hornuss et al. [13], who analyzed through a snapshot survey how this preventive tool has been adopted by the GBMSM community and what strategies are employed to access doxycycline. However, no such study has yet been conducted in Spain. In light of this, the objective of the current study, prepared and launched by the sexual health NGO “Stop Sida” from within and for the GBMSM community, is to analyse the use of doxycycline as a preventive tool among the GBMSM community in Spain.

## Methods

The NGO “Stop Sida” primarily focuses on the sexual health of lesbian, gay, trans, bisexual, queer, and other sexual minorities (LGTBQ+), as well as men and trans women who work as sex workers and chemsex users. “Stop Sida” mainly operates in Barcelona and Seville, but also provides online counselling services and has volunteers across the rest of Spain. To achieve the objective of the current study, the “Stop Sida” team replicated, using Microsoft Forms, the questionnaire from the beforementioned study by Hornuss et al. [13]. For that purpose, the team designed a communication campaign that included a visual poster that incorporated a link that lead to the questionnaire (poster provided as Supplementary Material 1). The poster was distributed as a snapshot survey in a snowball sampling manner through the NGO’s social networks (mainly *Instagram*) and various telegram groups (such as the one called *Chemsex Support* that gathers chemsex users) throughout February and March 2024.

The survey aimed to collect information about doxycycline use and included questions in four key areas: demographics (age range, location by postal code, origin, gender identity, and sexual orientation); STI history (diagnosis in the past year of syphilis, chlamydia, gonorrhoea, and other STIs); doxycycline history (whether participants had ever taken doxycycline before, whether it was taken for STI treatment, and whether it was taken as a preventive measure). For those who used doxycycline for prevention (PEP or PrEP), the survey included questions about how often they used it and how they obtained the medication. Descriptive statistics and hypothesis tests (mainly chi-square) were used for analysis, employing SPSS. The margin of error for survey responses was calculated with a standard deviation of 0.5 as described elsewhere [14]. A brief informed consent was obtained at the beginning of each questionnaire that included consent to participate and consent to publish. The participation to this study was not incentivized and all respondents answered the questionnaire voluntarily.

## Results

A total of 152 responses were collected, from which 150 were valid as 2 did not give consent. Forty-nine (32,7%) were from the Barcelona area, 10 (6,6%) from Madrid, 10 (6,6%) from Seville, while the rest 81 (54%) lived in different parts of Spain. 94% of respondents identified themselves as men, while 6% as non-binary; 95% were cis-gender while 5% were trans-gender; 87% defined themselves as homosexual, 9,6% as bisexual and the remaining 3,7% as other sexual orientation. 29% of the sample were people living with HIV (PLWH). 90% of HIV-negative respondents were on PrEP. Eighty-two respondents (54.6% of the total) had ever taken doxycycline, of whom 29 (35.4%) used it as Doxy-PEP and 7 (8.5%) as Doxy-PrEP. Of those taking Doxy-PEP, 5 (17.2%) did not follow the guidelines evaluated in the clinical trials (i.e. 200 mg within 72 h after condomless sex). Table 1 shows the intake of doxycycline per age range, and the number of doxy-PEP courses per year, indicating that more than half of doxy-PEP users makes 6 or less courses a year. There is a meaningful association among age range and doing doxy-PEP (chi-square = 149,00; p-value < 0,0001).

**Table 1:** Use of doxycycline of survey participants by age range

Age range	Has ever taken doxycycline, *n* (% of has ever taken doxycycline)	Doxy-PEP, *n* (% of doxy-PEP)	Number of doxy-PEP courses, *n* (% of doxy-PEP)					Doxy-PrEP, *n* (% of doxy-PrEP)
			1-2x year	2-6x year	1x month	2–3 x month	Every week	
18–25	2 (2.4)	-	-	-	-	-	-	1 (14.3)
26–30	7 (8.5)	1 (3.4)	-	1 (3.4)	-	-	-	1 (14.3)
31–35	14 (17.1)	5 (17.2)	1 (3.4)	4 (13.8)	-	-	-	-
36–40	22 (26.8)	9 (31.0)	-	6 (20.7)	2 (6.9)	1 (3.4)	-	2 (28.6)
41–45	15 (18.3)	4 (13.8)	-	-	1 (3.4)	1 (3.4)	2 (6.9)	2 (28.6)
46–50	9 (11.0)	4 (13.8)	-	2 (6.9)	-	1 (3.4)	1 (3.4)	1 (14.3)
50–55	10 (12.2)	5 (17.2)	2 (6.9)	-	3 (10.3)	-	-	-
56–60	1 (1.2)	-	-	-	-	-	-	-
60 +	2 (2.4)	1 (3.4)	-	-	-	1 (3.4)	-	-
Total, n (% of total)	82 (100)	29 (35.4)	3 (3.7)	13 (15.8)	6 (7.3)	4 (4.9)	3 (3.7)	7 (8.5)

Regarding the STI history shown in Table 2, doxy-PEP users showed higher self-reported frequency of not having been diagnosed in the last year of syphilis, chlamydia and gonorrhea compared to non-users. They also reported less frequency of being diagnosed once in the last year for all three STIs. However, they reported a higher frequency of having been diagnosed more than twice of chlamydia. Additionally, the association between Doxy-PEP use and a general decrease in self-reported diagnoses is significant only for chlamydia (chi-square test performed with df = 2 for all tests). It is important to note that these results should be interpreted with caution, as the data are self-reported, and factors such as the duration of Doxy-PEP use, dosage, adherence, and STI history were not verified.

**Table 2:** STIs history in the last year among doxy-PEP users and non-users

STIs diganosis in the last year		Doxy-PEP use		
		Yes, n (% of 29 cases)	No, n (% of 53 cases)	Chi-square (p-value)
Syphilis	None	25 (86,2)	35 (66,0)	3.8924 (0.1328)
	1x	3 (10,3)	13 (24,5)	
	2+	1 (3,4)	5 (9,4)	
Chlamydia	None	13 (44,8)	15 (28,3)	8.1749 (0.0167)*
	1x	7 (24,1)	30 (56,6)	
	2+	9 (31,0)	8 (15,1)	
Gonorrhea	Never	12 (41,4)	18 (34,0)	0.8943 (0.6394)
	1x	9 (31,0)	22 (41,5)	
	2+	8 (27,6)	13 (24,5)	

(*significant association)

**Fig. 1:**
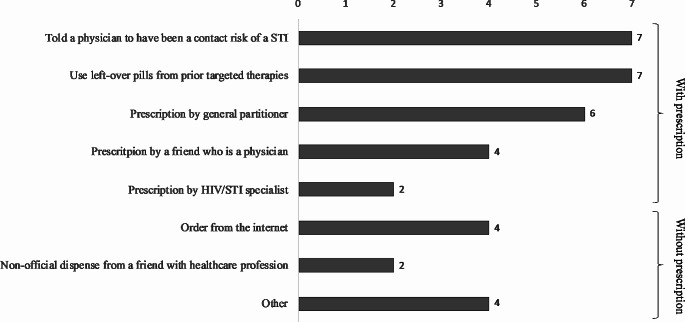
Modes of access to doxycycline for both PEP and PrEP use

The most common methods of getting doxycycline among individuals taking doxy-PEP or doxy-PrEP were telling a specialist that they have had a risky contact or using leftover pills from a previous treatment (both 7 cases, 19,4%), followed by obtaining a prescription by a general practitioner (6 cases, 17,7%). Four people (11,1%) got it by prescription by a friend who is physician, and other 4 purchased it online. In total, 22 individuals (72,2%) got the doxycycline through a method involving a medical prescription in some way, while the remaining 10 cases (27,8%) avoided the prescription (e.g., by ordering it online, or non-official channels). Among the 4 respondents who selected “others”, 2 obtained the doxycycline from a sexual health organization, 1 was a doctor who self-prescribed it, and 1 purchased it illegally to a “doctor who is a dealer”. Fig. 1 illustrates these results.

## Discussion

The present study replicated the methodology used in [13], incorporating additional questions concerning STI history. The findings are consistent with those observed in Germany: a comparable proportion of GBMSM community members sampled had taken doxycycline in the past year (54.6% in Spain vs. 55% in Germany) and were using it as Doxy-PEP (35.4% vs. 23%) or Doxy-PrEP (8.5% vs. 6%). The methods for obtaining doxycycline were also strikingly similar, with the use of leftover pills from previous treatments being the most common strategy in both contexts. However, while this approach was as prevalent as informing a doctor of a risky STI contact in Spain, it was almost negligible in Germany. Additionally, the study revealed that Doxy-PEP use correlated with a decreased frequency of being diagnosed with syphilis, chlamydia, and gonorrhea in the past year, although in some cases, the frequency of diagnosis increased. Notably, the association between Doxy-PEP use and a general decrease in diagnoses was significant only for chlamydia. Nonetheless, these findings should be interpreted cautiously as they rely on self-reported data, and factors such as duration, dosage, and adherence to Doxy-PEP were not adequately assessed, crucial for understanding the association between Doxy-PEP use and STI incidence. Nevertheless, both studies indicate that a substantial and comparable portion of the GBMSM community in Spain and Germany is already integrating doxycycline into their sexual health prevention strategies.

This study provides the first description of how doxycycline is being incorporated as a prevention strategy by the GBMSM community in Spain. Moreover, it is the inaugural research effort of its kind initiated from within the GBMSM community itself, designed, executed, and analyzed by the NGO “Stop Sida.” Acknowledging the limitations of a snapshot questionnaire survey, it is important to recognize that the data are not sufficiently representative, necessitating more robust methodological approaches to accurately assess the extent of doxycycline use as a prevention strategy in the GBMSM community in Spain. Furthermore, it must be emphasized that the sample cannot be deemed representative of the GBMSM community, given that recruitment was conducted using a snowball strategy within “Stop Sida” social networks. Despite these limitations, the data suggest that, although medical and scientific associations seldom endorse community doxy-PEP use as a prevention strategy, the GBMSM community is integrating it as a preventive measure. However, this lack of regulation and decision-making may result in the misuse of doxycycline as a preventive strategy in regimes lacking robust evidence (e.g., doxy-PrEP or alternative doxy-PEP guidelines) or the acquisition of doxycycline through unregulated channels or by providing false information to healthcare professionals. Thus, this study underscores the urgent need for a medical and scientific discourse that, in collaboration with civil society entities engaged in sexual health, formulates evidence-based and systematic guidelines for this preventive tool, ensuring the quality of treatments and the training of professionals responsible for recommending it.

The utilization of doxycycline as a prevention strategy warrants a thorough evaluation of its impact on the development of resistance among targeted pathogens and other STI agents such as *Mycoplasma genitalium* [15] or other non-STI related agents such as *Staphylococcus aureus, Escherichia coli* or *Haemophilus influenzae* [16]. Recent studies have also discussed additional effects of doxycycline on the development of resistance in *N. gonorrhoeae* against ceftriaxone, the current first-line treatment in gonorrhea [17]. Nevertheless, Traeger et al. [18] demonstrated through a modeling study analyzing data from an LGBTQ-focused health center in the US that prescribing doxy-PEP based on STI history resulted in an efficient strategy balancing uptake and preventive impact. Moreover, the Doxy-PEP study [2] showed a low resistance effect among non-STI agents and on *T. palladium* and *C. trachomatis.* All these considerations should be taken into account to assess the long-term implications of implementing doxy-PEP strategies.

## Electronic supplementary material

Below is the link to the electronic supplementary material.

Supplementary Material 1

## Data Availability

Access of the snapshot survey dataset can be provided on individual request.
