# Correction: A practice already in use: a snapshot survey on the use of doxycycline as a preventive strategy (Doxy-PEP and Doxy-PrEP) in the GBMSM population in Spain

**DOI:** 10.1007/s15010-025-02514-y

**Published:** 2025-03-19

**Authors:** Sergio Villanueva Baselga, Ruben Mora, Luis Villegas

**Affiliations:** 1 Stop Sida, Barcelona, Spain; 2 Centre of Research for Information, Communication and Culture (CRICC), University of Barcelona, Barcelona, Spain

**Correction to: Infection (2024) 53:437–441**

10.1007/s15010-024-02320-y

In the original version of this article, the given and family names of Sergio Villanueva Baselga, Ruben Mora and Luis Villegas were incorrectly structured. The name was displayed correctly in all versions at the time of publication.

Villanueva Baselga, Sergio, Mora, Ruben, Villegas, Luis

The name is Sergio, and the 2 surnames Villanueva Baselga No hyphen between my 2 surnames.

Sergio Villanueva Baselga

