# Supplementary figures and images for: A practice already in use: a snapshot survey on the use of doxycycline as a preventive strategy (Doxy-PEP and Doxy-PrEP) in the GBMSM population in Spain
Source: Infection. 2024 Jul 25;53(1):437–41. doi: 10.1007/s15010-024-02320-y (PMC11825527; doi:10.1007/s15010-024-02320-y)

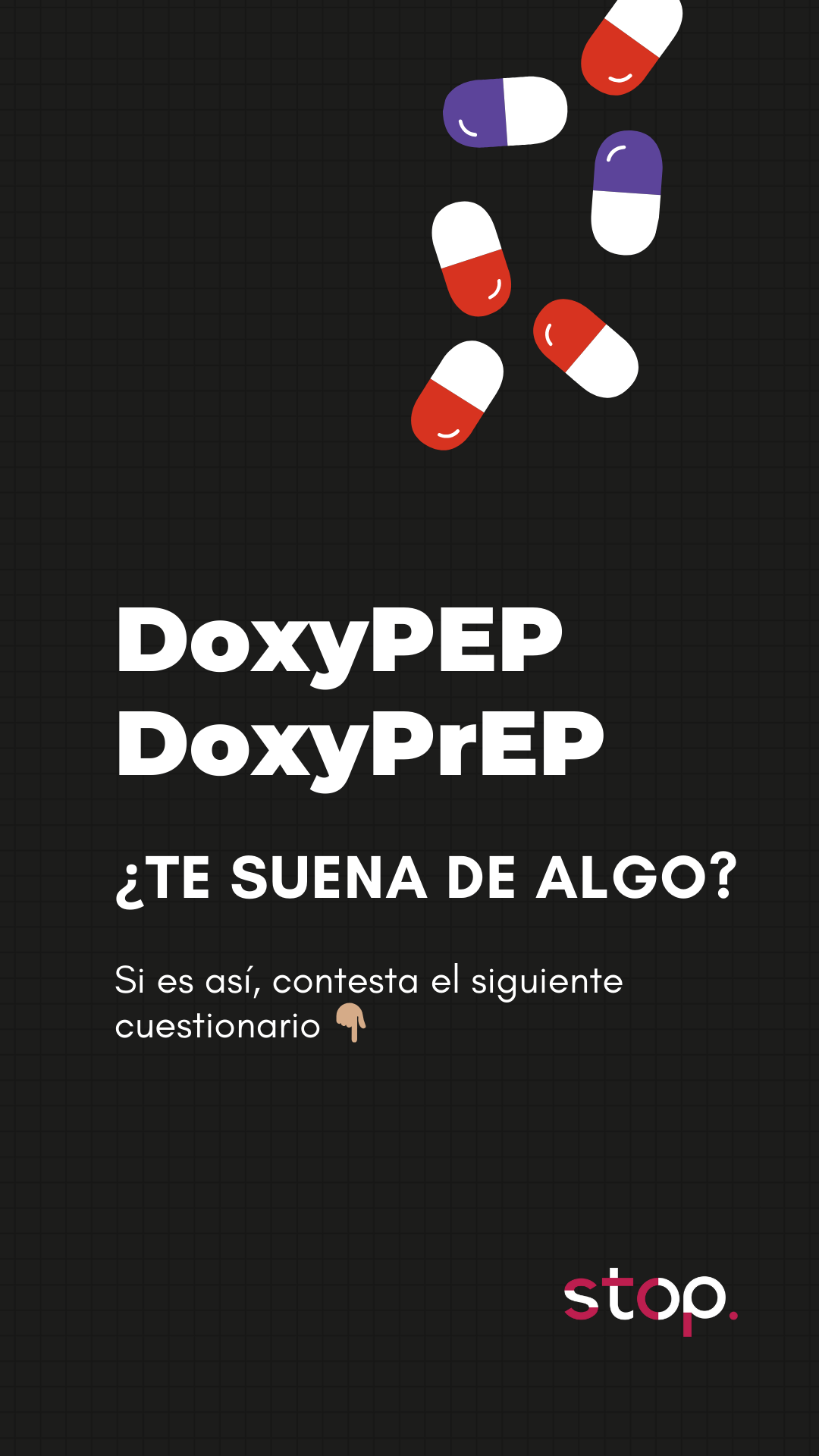

Supplement: Supplementary file 1 — Supplementary Material 1 [file 15010_2024_2320_MOESM1_ESM.png]
